# Effects of Nutritional Interventions on Athletic Performance

**DOI:** 10.3390/nu15214498

**Published:** 2023-10-24

**Authors:** Ampe Toon, Stephen Bailey, Bart Roelands

**Affiliations:** 1 Human Physiology & Sport Physiotherapy Research Group, Vrije Universiteit Brussel, 1050 Ixelles, Belgium; toon.ampe@vub.be; 2 Department of Physical Therapy Education, Elon University, Elon, NC 27244, USA; baileys@elon.edu

The search to comprehend the fundamental physiological factors that contribute to the exceptional endurance performance of elite human athletes is a long-standing endeavor within the field of sports science research [1]. Even today, this search remains a central focus. Currently, a significant portion of research is aimed at unravelling the role of nutritional interventions in athletic performance. As stated in the IOC consensus statement, athletes use dietary supplementation (among others) for supporting their energy metabolism, correcting deficiencies, and achieving either direct or indirect ergogenic effects [2]. There are many systematic reviews [3,4,5,6,7] and consensus statements [2,8,9,10,11] on athletes’ dietary supplementation, especially for macronutrients (e.g., glucose, protein, and ketone supplementation, among others) and micronutrients (e.g., nitrate, vitamin, caffeine, and electrolyte supplementation, among others). Therefore this editorial is focused on the emerging, but yet to be further researched, supplementation with substances (i.e., probiotics, prebiotics, and short-chain fatty acids (SCFAs)) that affect the gut microbiome. The gastrointestinal tract (GIT) is inhabited by trillions of not only bacteria, but also viruses, fungi, and archaea [12]. With approximately nine million genes [13], this environment represents the human gut microbiome, which makes up a complex ecological community by which an individual lives in a symbiotic manner all his life [14]. The gut microbiome has versatile functions, such as energy production, regulating the immune system, producing hormones, and communicating with the brain [8,15]. This has ensured that, in the last ten years, a significant interest in understanding the connection between exercise and the gut microbiome has arisen.

Emerging from preliminary observations in both animal and human models, interventions that focus on optimizing the microbial balance have showcased improvements in exercise capacity. Consequently, it is becoming evident that the gut microbiota might constitute an untapped factor in influencing exercise performance. Strategies that externally target the gut microbiota offer a new opportunity for augmenting human exercise tolerance, capacity, and overall performance. This editorial therefore aims to uncover the ways in which the gut microbiome could be harnessed (i.e., with probiotics, prebiotics, and SCFAs) to improve exercise tolerance and capacity.

Prebiotics and probiotics comprise a family of substances that modulate the gut microbiota and collectively can be referred to as ‘biotics’ [16]. The utilization of biotics to augment the abundance of beneficial bacterial species (probiotics) or to nourish the existing commensal bacterial species (prebiotics) represents an exogenous approach to enhance the structure and function of the gut microbiota [17]. Probiotics are defined as “live microorganisms that confer a health benefit to the host when administered in adequate amounts”. They are mostly administered in the form of singular, or blends of, Lactobacillus and Bifidobacterium strains [18,19].

Studies from 2019 to 2022 have demonstrated that supplementing mice with various single bacteria strains led to an improved endurance exercise capacity [20,21,22,23,24,25,26], likely through their effect on the energy metabolism, intestinal barrier integrity, and overall host health [27,28,29,30]. This is further supported by the position stand of the International Society of Sports Nutrition, which affirms that probiotic supplementation can mitigate an increased gut permeability resulting from intense, prolonged exercise, particularly in hot conditions [31].

To this day, human studies on probiotic supplementation in general mostly use commercially available probiotics and not a specifically targeted strain of bacteria, as in the above-mentioned mice studies. However, a review on these human studies also showed a positive effect on aerobic performance in trained individuals [32]. According to the same study, a supplementation period of less than 4 weeks with a single-strain probiotic might give the most promising results. Aerobic performance is the most studied parameter so far; nevertheless, an increased anaerobic performance among mixed martial arts athletes was also found when combining probiotics with vitamin D3 [33].

Within athletic populations, specific strains of probiotics have the potential to enhance the absorption of essential nutrients such as amino acids from protein, consequently influencing the pharmacological and physiological attributes of various food constituents [31]. However, according to another review, too little is known about the effects of the gut microbiome, and, therefore, about biotic supplementation, with regard to the ergogenic effects of most dietary supplements [34]. Lastly, the addition of probiotics to the diet has been shown to decrease the occurrence, duration, and intensity of upper respiratory tract infections, potentially leading to indirect enhancements in training or competitive performance [6,35]. Following this, an improved recovery from muscle damage is shown during probiotic supplementation [31]. However, the impact of variations in the gut microbiota composition on the effectiveness of probiotics remains uncertain [31].

In summary, a moderate number of direct and indirect ergogenic effects of probiotics have been discovered, making probiotic supplementation a promising ergogenic intervention for athletes. However, more specific research is needed in consideration of specific bacterial strains, different populations, different types of physical performance, and dose–response relationships, as well as their effect on other dietary supplementations. 

A prebiotic is a substance that is selectively metabolized by indigenous microorganisms in the host, providing a health advantage. It is present in non-digestible compounds such as oligosaccharides, galactans, and fructans [36]. A whole list of potential fiber and prebiotic types, as well as their effect on host physiology, can be found in the study of Bongiovanni and colleagues [37]. The consumption of particular dietary components, namely prebiotics and fiber, is widely recognized for conferring numerous health benefits [38]. Dietary fibers, in addition to promoting bowel regularity, play a vital role in maintaining gut health through a diverse array of phytochemicals and metabolites, including SCFAs [39,40]. This underscores the importance of an adequate fiber intake for everyone, including athletes. Individual tolerance to fiber and prebiotics varies. Modest reductions in the consumption of fiber-rich foods may be due, in part, to discomforts such as bloating, cramps, and increased flatulence that are experienced post-fiber consumption [41]. This is a crucial consideration when devising nutritional plans for athletes, particularly when aiming to boost fiber intake, and especially during competitions. Because of these possible discomforts, athletes often follow diets that are high in carbohydrates and protein, but with a lower fiber intake, matching their energy needs for training and competitions. A reduced fiber intake shifts microbial fermentation towards less-favorable substrates such as proteins and fats, decreasing SCFA production [42,43]. Notably, just a brief 24 h shift in the macronutrient balance can alter the microbial community, influencing the preference for carbohydrates over proteins [44,45]. Furthermore, a study in mice with both high and low levels of accessible, non-fermentable carbohydrates for the microbiota showed shorter run times in the low-level accessible group [46]. These effects were reversed when those mice (low) received a prebiotic combined with fecal transplantation from the other group (high). This study highlights the important role of prebiotics (and food strategy) in physical performance. Subsequent research should be structured to investigate the augmentation of circulating SCFAs through prebiotic and fiber consumption in relation to exercise performance and recovery, taking in account the possible GIT discomforts [37]. 

The use of postbiotics has surfaced more recently, and is characterized as a “preparation of inanimate microorganisms and/or their components that confers a health benefit on the host” [47]. Postbiotics encompass deactivated microbial cells or cellular constituents, potentially accompanied by metabolites such as SCFAs [48]. It should be noted that the accessibility of such supplements in the commercial market is upcoming, but still limited. However, the isolated supplementation of SCFAs in a physical performance context is already more investigated.

Bacterial fermentation yields SCFAs such as acetate, propionate, and butyrate. Their production ratio is approximately 60:20:20, but this can vary [49,50]. SCFA levels peak in the proximal colon (70–140 mM), whilst decreasing in the distal colon (20–70 mM). Around 95% of SCFAs are absorbed by colon cells, with the remaining 5% being excreted in feces [49,50]. Butyrate is the main energy source for colon cells, promoting intestinal health [51]. Acetate and propionate are also used, but to a lesser extent [52]. Unused SCFAs travel to the liver, where they can be used for energy or enter the bloodstream, benefiting various organs. Recent findings suggest that SCFAs may influence the skeletal muscle metabolism via specific receptors (GPR41 and GPR43) [53,54]. This is significant for understanding the exercise-related energy metabolism.

As further investigated by Scheiman and colleagues, a rectal supplementation with propionate increased running times in mice during a time to exhaustion (TTE) test compared to saline supplementation [25]. Additionally, infusing acetate into microbiota-depleted mice improved treadmill run times, emphasizing its role in exercise performance [46]. These studies show that acetate and propionate have restorative and potentially performance-enhancing effects. Additionally, research on mice lacking Acetyl-CoA synthetase 2 confirms that microbiota-generated acetate is crucial for the skeletal muscle metabolism and may influence exercise tolerance [55]. Acetate availability to tissues with SCFA receptors, including the skeletal muscles, suggests that interventions that involve increasing acetate bioavailability could improve exercise tolerance. In humans, SCFAs have been linked to the stimulation of mitochondrial biogenesis and the promotion of endurance performance [56]. Hence, elevating the presence of SCFAs in the system could be a novel approach to enhance exercise capacity, offering an extra energy source to active tissues. However, the impact under various pre-exercise feeding conditions is unclear [19]. 

To conclude, further investigation is required to understand the specific direct or indirect mechanisms, as well as the potential interactions, through which gut-derived SCFAs might impact exercise performance.
